# Anti-fibrotic effect of *Holothuria arenicola* extract against bile duct ligation in rats

**DOI:** 10.1186/s12906-015-0533-7

**Published:** 2015-02-05

**Authors:** Sohair R Fahmy

**Affiliations:** Department of Zoology, Faculty of Science, Cairo University, 12613 Giza, Egypt

**Keywords:** *Holothuria arenicola*, Antifibrotic, Antioxidant, Bile duct ligation, Cholestatic indices

## Abstract

**Background:**

*Holothuria arenicola* is the most important and abundant sea cucumber species in the Mediterranean Sea on the Egyptian coast. The present study aims to assess the anti-oxidative and anticholestatic effects of the sea cucumber *Holothuria arenicola* extract (HaE) in a model of bile duct ligation in male albino rats.

**Methods:**

Fifty four male Wistar albino rats were assigned into two main groups, the Sham-operated control and bile duct ligated (BDL) group. After 14 days of surgery, the animals of the group I (Sham control) received distilled water only for 7, 14 and 28 days. Second group (BDL group) was divided into 2 subgroups, animals of these subgroups treated for 7, 14 and 28 consecutive days as follow: subgroup I (BDL), rats of this subgroup administered distilled water orally. Subgroup II (HaE), animals of this subgroup treated orally with HaE (200 mg/kg body weight).

**Results:**

The HaE revealed significant antifibrotic effect as evident by decreasing the levels of total conjugated and unconjugated bilirubin and the activities of serum aminotransferases (ASAT and ALAT) and alkaline phosphatase (ALP) as well as malondialdehyde (MDA) level, and increasing the serum albumin, glutathione reduced (GSH) levels. Treatment with HaE normalized the antioxidant enzyme, glutathione-S-transferase (GST), superoxide dismutase (SOD) and catalase (CAT) activities activities.

**Conclusion:**

The present prospective study correlated the antifibrotic effect of HaE to its direct antioxidant effect that can be related to its contents of phenolic compounds specially chlorogenic acid, pyrogallol, rutin and coumaric acid.

## Background

Cholestasis represents the consequence of impaired bile formation and generally classified as extra- and intrahepatic [1]. Cholestasis is a reduction in bile flow that leads to the intrahepatic accumulation of bile acids and other toxic compounds with progression of liver pathology, including hepatocellular injury and fibrosis [2]. The failure of bile salts excretion in cholestasis leads to retention of hydrophobic bile salts within the hepatocytes causing apoptosis and necrosis [3]. Moreover, abnormal flux of bile acids and bilirubin in the liver leads to retention and accumulation of toxic hydrophobic bile salts within hepatocytes [4], causing inflammatory reactions, hepatocyte death, and periductular fibrosis [5]. Recent studies have demonstrated that inflammatory injuries and oxidative stress occur in the liver with cholestasis [6,7].

Oxidative stress has been implicated in the process of fibrogenesis [8,9]. Cholestatic liver fibrosis, characterized by excessive accumulation of extracellular matrix (ECM) proteins, is associated with bile acid-induced oxidative stress and lipid peroxidation [10]. Furthermore, oxidative stress aggravates liver fibrosis via stellate cell activation [11]. Bile duct ligation (BDL) is a typical model of biliary cholestasis in animals [12], that induced oxidative damage and fibrosis in rats [13]. Moreover, the bile salts are partly responsible for the plasma membrane damage seen in the bile-duct ligated models that leads to further oxidative stress [14,15].

The problems associated with antifibrotic drugs are chronic administration, the reduced therapeutic effects and toxicity, so developing antifibrotics from natural products may reduce the risk of toxicity and maintain the therapeutic effectiveness when they used clinically [16]. As a consequence of an increasing demand for the therapeutic drugs, products from marine sources have become attractive as nutraceutical and functional foods and as a source material for the development of drugs [17]. Sea cucumber *Holothuria* is a gelatinous marine resource that is shaped as a cucumber. It is considered “sea ginseng” because of its known medicinal properties aside from its nutritional value. The therapeutic use of sea cucumbers for healing is established, where they used for joint pain, tendonitis and sprains [18]. Another report has demonstrated the antinociceptive property of gamat (*Holothuria* spp) [19]. Sea cucumber is also remarkably rich in vitamins, trace elements, and polysaccharides (condroitin sulfate), that reduce arthritis pain and inhibit viral activities, and saponin glycosides that inhibit cancer activities [20]. Recently, Esmat et al. [21] revealed that the sea cucumber mixed extract contains physiologically active phenolic compounds with antioxidant activity that afforded a potential hepatoprotective activity against thioacetamide induced liver injury in a rat model.

*Holothuria arenicola* is the most important and abundant sea cucumber species in the Mediterranean Sea on the Egyptian coast [22]. The present study aims to assess the anticholestatic effects of the sea cucumber *Holothuria arenicola* extract (HaE) in a model of bile duct ligation in male albino rats and to elicit the underlying *in vivo* anti-oxidative mechanisms.

## Methods

### Sample collection

Sea cucumbers (*Holothuria arenicola)* were collected from the Abu – Qir Bay in the Egyptian Mediterranean coast of the eastern Alexandrian coast (May-June 2012). The animals were transported to our laboratory in an ice box containing ice cubes and a few pinches of table salt. The animals were immediately washed under running tap water and cut open, and all visceral organs were removed and then the body walls of the animals were stored at ^_^20°C until processing.

### Preparation of the *Holothuria arenicola* extract

The phosphate buffer extract was prepared according to the method of Yasumoto et al. [23]. The body wall of the animals was cut into small parts and blended in phosphate buffer (in a volume = 4 ml X tissue weight) and extracted at room- temperature (25°C) with pH 7.2 for 5 hours. The filtered was collected immediately, concentrated and lyophilized using lyophilizer (LABCONCO lyophilizer, shell freeze system, USA).

### Antioxidant activity

#### Free radical scavenging activity

The free radical scavenging activities of the extract and ascorbic acid were analyzed by the DPPH assay [24]. A 1.0 ml of the test extract, at gradient final concentrations of 10 - 80 mg/ml, was mixed with 2 ml of 0.3 mM DPPH solution in MeOH in a cuvette. The absorbance was taken at 517 nm after 20 minutes of incubation in the dark at room temperature. The experiment was done in triplicates. The percentage antioxidant activity was calculated as follows:

- %Antioxidant Activity [AA] = 100 - [{(Abs_sample_ – Abs_blank_) X 100}/Abs_control_]. Where Abs_sample_ was the absorbance of sample solution (2.0 ml) + DPPH solution (1.0 ml, 0.3 mM), Abs_blank_ was the absorbance of Methanol (1.0 ml) + sample solution (2.0 ml), Abs_control_ was the absorbance of DPPH solution (1.0 ml, 0.3 mM) + methanol (2.0 ml).

### High-performance liquid chromatography analysis

The phenolic components of sea cucumber extract were separated by high performance liquid chromatography using an Agilent 1100 device (Waldborn, Germany) equipped with a Zorbax reversed-phase 300SB C18 column (250–4.6 mm) with 5-mm particle size (Lawrence, KS, USA) and ultraviolet detector (G1314A) adjusted at 280 NM. Sample and authentic standards (50 mL; chlorogenic acid, coumaric acid, catechin, ascorbic acid, pyrogallol, and rutin) dissolved in dimethyl sulfoxide and acidified with a drop of acetic acid; then they injected onto the column. The mobile phase was 0.4% formic acid and acetonitrile (60:40, v/v) with a constant flow rate of 1 ml/min. The isolated peaks of the phenolic compounds in the sample were identified by comparing their relative retention times with those of the standards, and then the concentration (percentage) of each compound was calculated as peak area integration.

### Ethical consideration

Experimental protocols and procedures used in this study were approved by the Cairo University, Faculty of Science Institutional Animal Care and Use Committee (IACUC) (Egypt), (CUFS/F/06/13). All the experimental procedures were carried out in accordance with international guidelines for the care and use of laboratory animals.

### Experimental animals

The experimental animals used in this study were male Wistar rats (*Rattus norvegicus*) weighing 150–160 ± 5 g. The animals were obtained from the National Research Center (NRC, Dokki, Giza). Animals were grouped and housed in polyacrylic cages (six animals per cage) in the well– ventilated animal house of the Department of Zoology, Faculty of Science, Cairo University. Animals were given food and water *ad libitum*. Rats were maintained in a friendly environment with a 12 h/12 h light–dark cycle at room temperature (22°C–25°C). Rats were acclimatized to laboratory conditions for 7 days before commencement of the experiment.

### Component of diet

The diet containing (~22% protein, ~3.5% fats, ~ 0.25% vitamins, ~ 0.5% sodium chloride, ~ 0.72% molasses, ~ 60% maize, ~ 20% soybeans, ~ 10% concentrate fattening, and ~ 5% bran).

### Toxicity study (OECD 420)

Wistar rats weighing (150–160 g) were used for acute toxicity study. The animals (12 rats) were divided into control and test groups containing six animals each. The rats were administered orally with sea cucumbers *Holothuria arenicola* extract (HaE) at dose levels of 5 g/kg (high dosage) and 2 g/kg (low dosage). Normal control rats received the same amount of vehicle (distilled water) only. Animals were observed carefully for 24 hours after extract administration and then for the next 14 days. At the end of this experimental period, the rats were observed for signs of toxicity, morphological behavior, and mortality. Acute toxicity was evaluated based on the number of deaths (if any). Acute toxicity was calculated as OECD guidelines 420 (Fixed dose method) [25,26].

### Bile duct ligation induced liver damage

Bile duct ligation performed according to Vogel and Vogel [27]. Rats were anesthetized with ketamine and chlorpromazine (100 mg/kg ketamine and 0.75 mg/kg chlorpromazine; ip). Laparotomy was performed under antiseptic conditions. A mid-line incision in the abdomen was made, exposing the muscle layers and the linea alba, that was then incised over a length corresponding to the skin incision. The edge of the liver was then raised and the duodenum pulled down to expose the common bile duct, which pursues an almost straight course of about 3 cm from the hilum of the liver to its opening into the duodenum. There is no gall bladder, and the duct was embedded for the greater part of its length in the pancreas, which opens into it by numerous small ducts. A blunt aneurysm needle was passed under the part of the duct selected, stripping the pancreas away with care, and the duct was divided between double ligatures of cotton thread. The peritoneum and the muscle layers as well as the skin wound were closed with cotton stitches. In sham-operated rats, abdominal incision was made without a bile duct ligation.

### Experimental design

Fifty four male Wistar rats were assigned into two main groups, the Sham-operated control (18 rats/group) and bile duct ligated (BDL) group (36 rats/group). The bile ducts of animals of group II were ligated. After 14 days of surgery, the animals of group I received distilled water only for 28 days. The second group was divided into 2 subgroups (18 rats/subgroup), animals of these subgroups treated for 28 consecutive days as follows:

#### Subgroup I (BDL)

Rats of this subgroup administered distilled water orally.

#### Subgroup II (HaE)

Animals of this subgroup treated orally with HaE (200 mg/kg body weight).

### Animals handling

Animals were euthanized on the 8th, 15th and 29^th^days of treatment after being fasted overnight under deep anesthesia with ketamine and chlorpromazine. Blood collected by cardiac puncture. Blood was collected in centrifuge tubes. Liver was removed and immediately blotted using filter paper to remove traces of blood and then divided into two parts, the first part stored at −80°C for biochemical analysis. While, the second part was suspended in 10% formal saline for fixation and preparation to histological processing.

### Sample preparation

#### Serum preparation

Blood samples collected in centrifuge tubes were centrifuged at 3000 rpm for 20 minutes. Serum stored at −20°C until used for biochemical assays.

### Liver homogenate preparation

Liver tissue was homogenized (10% w/v) in ice-cold 0.1 M Tris–HCl buffer (pH 7.4). The homogenate was centrifuged at 3000 rpm for 15 min. at 4°C and the resultant supernatant used for biochemical analysis.

### Histopathological study

Liver slices were fixed in 10% formal saline and embedded in paraffin wax blocks. Sections of 5 μm thick were stained with hematoxylin & eosin (H&E) then examined under light microscope for determination of pathological changes [28]. Picrosirius Red stains of paraffin-embedded sections were used to qualitatively assess collagen architecture and extent of fibrosis. Morphometric analysis for fibrosis quantification was performed using 10 photographs of random high-power fields (200×) for each liver sample. These images were analyzed with LIVARQ500 software (Pathology department of National Research Center).

### Biochemical analysis

#### Serum biomarkers for liver function tests and total protein level

The appropriate kits (Bio-Diagnostic, Dokki, Giza, Egypt) was used for the determination of total protein by colorimetric method according to the method described by Tietz [29]. Albumin is determined using a calorimetric end point method according to modified bromocresol green binding assay (BCG), Tietz [30]. Serum aminotransferase enzyme activities (ASAT & ALAT) were determined according to Reitman and Frankel [31]. All assays were made according to the instructions of the manufacturer.

### Serum cholestatic indices

Alkaline phosphatase was assayed by the appropriate kits (Bio-Diagnostic, Dokki, Giza, Egypt) according to the method of Belfield [32]. Total bilirubin and direct bilirubin were measured by the method of Walter and Gerade [33]. Indirect bilirubin was calculated as follows:

$$ \mathrm{Indirect}\ \mathrm{bilirubin}=\mathrm{Total}\ \mathrm{bilirubin}-\mathrm{direct}\ \mathrm{bilirubin} $$

### Oxidative stress markers assessment

Oxidative stress markers were detected in the resultant supernatant of liver homogenate. The appropriate kits (Biodiagnostic kits, Biodiagnostic Dokki, Giza, Egypt) were used for the determination of malondialdehyde (MDA) [34], glutathione reduced (GSH) [35], catalase (CAT) [36], glutathione-S-transferase (GST) [37] and superoxide dismutase (SOD) [38]. All assays were made according to the instructions of the manufacturer.

### Statistical analysis

Values were expressed as means ± SE. To evaluate differences between the groups studied, one way analysis of variance (ANOVA) with the Duncan post hoc test was used to compare the group means and *P* < 0.05 was considered statistically significant. SPSS for Windows (version 15.0) was used for the statistical analysis.

% improvement = treated mean - injured mean/control mean × 100.

## Results

### Acute toxicity

From the experiment performed as per the OECD Guidelines 420, the results reveal that the *Holothuria arenicola* (HaE), has been found toxic at 5000 mg/kg body weight of experimental animals as in the first 4 hours of observation2/3 morbidity was observed. None of the 6 rats died or showed any sign of toxicity at the limit dose of 2000 mg/kg p.o. for HaE in the first 48 h. No evidence of toxicity was noticed during the period of observation. The LD_50_ was therefore taken as above 2000 mg/kg p.o. The median effective dose (ED_50_) was selected based on the proposed LD_50_ obtained from the acute toxicity study. This dose was considered one tenth of the proposed LD_50_, that is, 200 mg/kg body weight.

### Free radical scavenging activity

**Figure 1:**
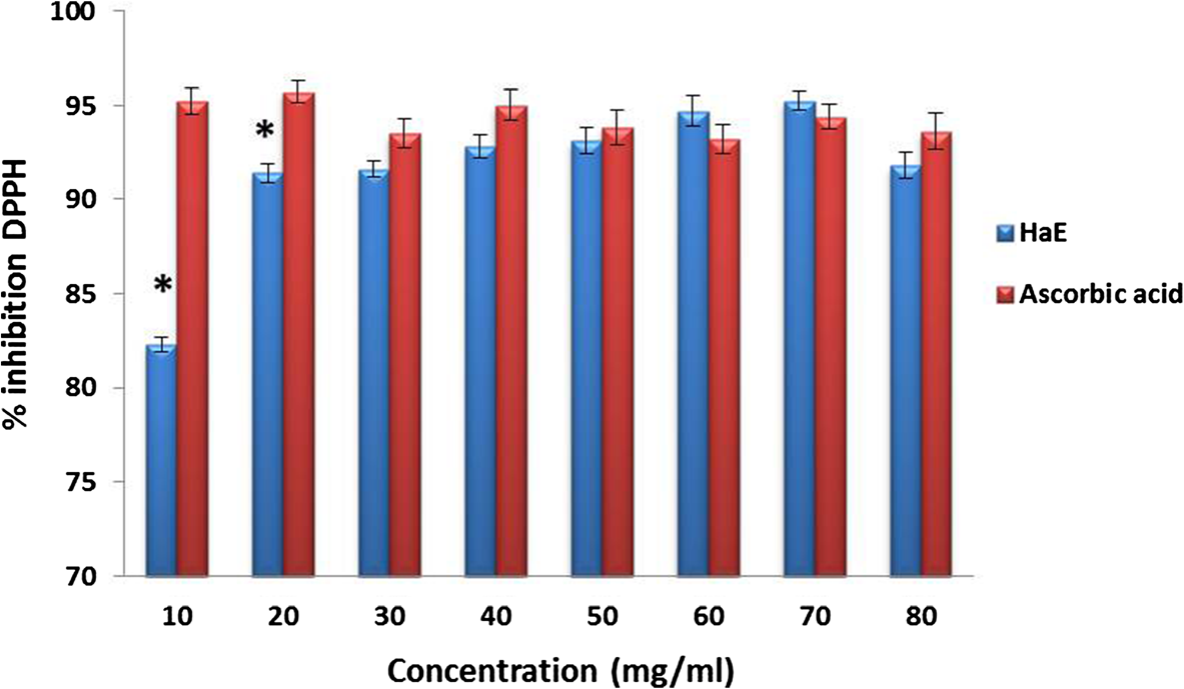
**Antioxidant activity of** ***Holothuria arenicola*** **extract (HaE) and ascorbic acid.** Each vertical column represents the mean ± SEM of change of 6 mice. *: significant at p < 0.05 as compared to the ascorbic acid.

The results of DPPH scavenging activity of HaE and ascorbic acid were shown in the Figure 1. The radical scavenging activities were estimated by comparing the percentage of inhibition of DPPH radical to the tested extract and the ascorbic acid. The data were displayed with mean ± SEM of three replications. The present results showed that HaE produced dose dependent inhibition of DPPH radical ranging from (82.3 to 95.2%) as compared to ascorbic acid.

### Phenolic compounds in the *Holothuria arenicola* extract

**Figure 2:**
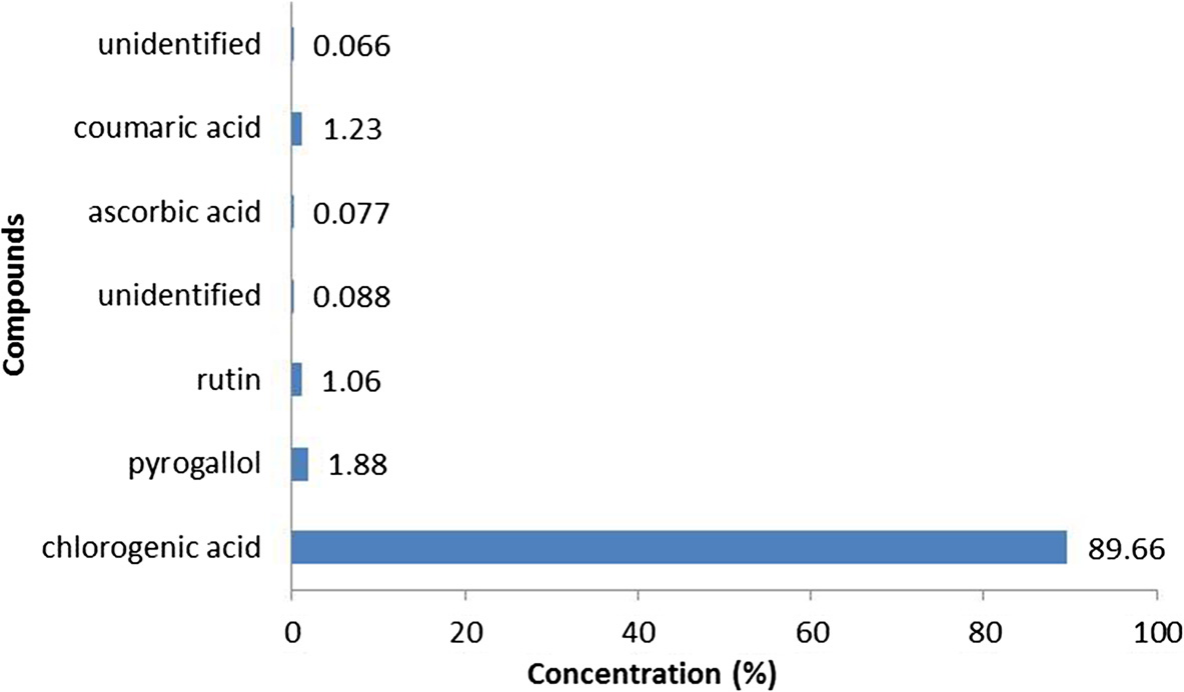
**High-performance liquid chromatographic analysis of** ***Holothuria arenicola*** **extract.**

High-performance liquid chromatography analysis of HaE revealed the presence of seven non-volatile phenolic compounds, two of which were unidentified under the adopted conditions. Chlorogenic acid was the major component (89.66%), whereas ascorbic acid (0.077%) was the minor component. Other components, such as pyrogallol (1.88%), rutin (1.06%) and coumaric acid (1.23%) were recorded in Figure 2.

### Liver histopathological analysis

**Figure 3:**
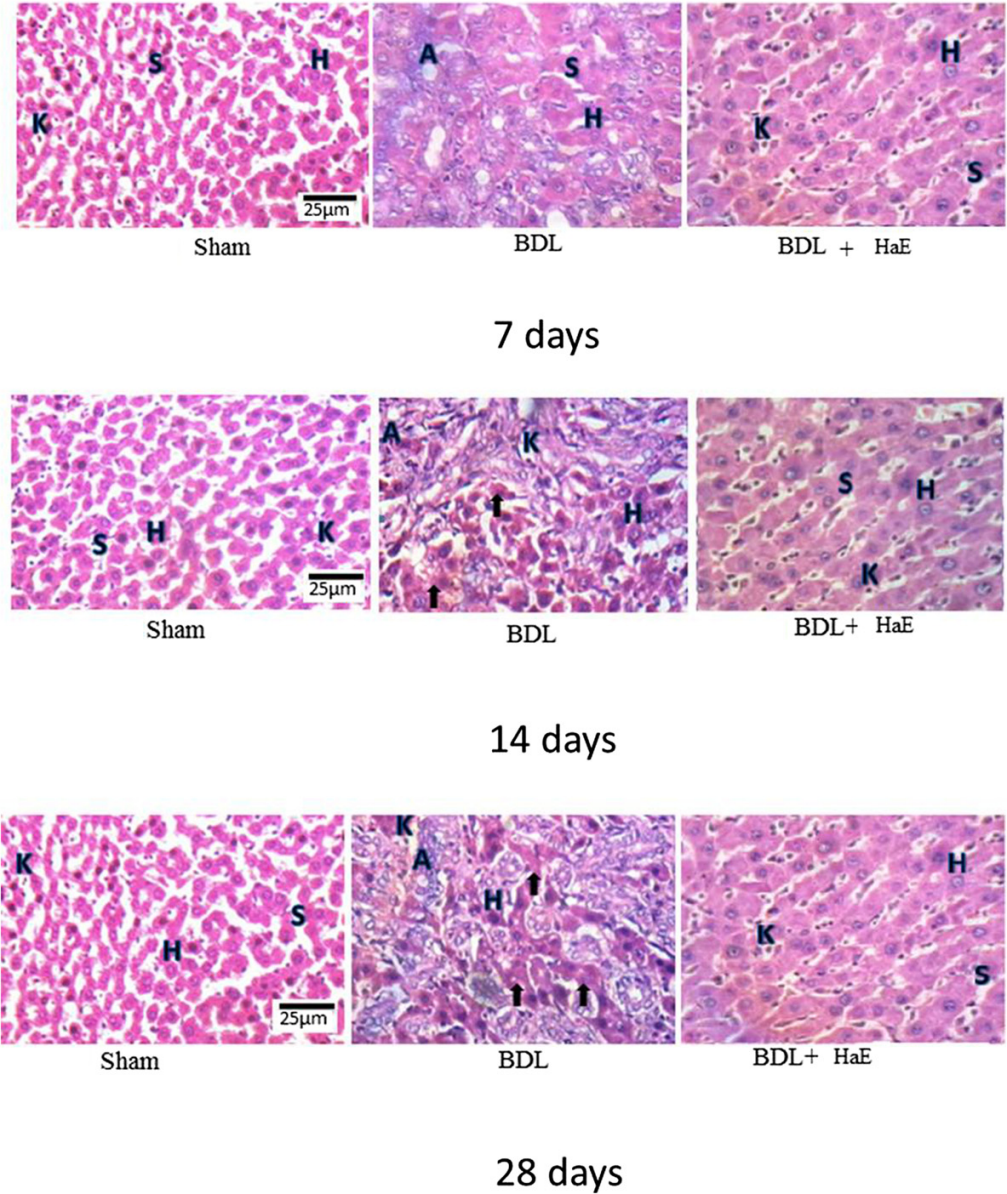
**Histological study of hematoxylin& eosin stained liver sections (400×) of Sham, BDL and** ***Holothuria arenicola*** **extract (HaE) treated rats.** Sham treated liver showing normal hepatocytes (H) and separated by blood sinusoids (S). Bile duct ligated liver showing liver fibrosis indicated by adipocytes (A) and many collagen fibers (arrows). HaE- treated liver sections demonstrating the regeneration of liver parenchyma and light fibrosis.

Representative views of liver sections are shown in Figure 3. As shown in tissue sections stained with hematoxylin and eosin, compared with liver sections of the Sham group, BDL caused prominent proliferation of Kupffer cells (K), abundant adipocytes (A) and marked deposition of collagen fibers (arrow). For a long time period (28 days), liver sections showed necrosis, apoptosis and abundant marked deposition of collagen fibers leading to loss of hepatic lobular architecture. Oral administration of HaE daily for 14 and 28 days improved the hepatic architecture; as compared to the corresponding BDL group and it apparently suppressed hepatic fibrogenesis by reducing the thickness of bridging fibrotic septa.

**Figure 4:**
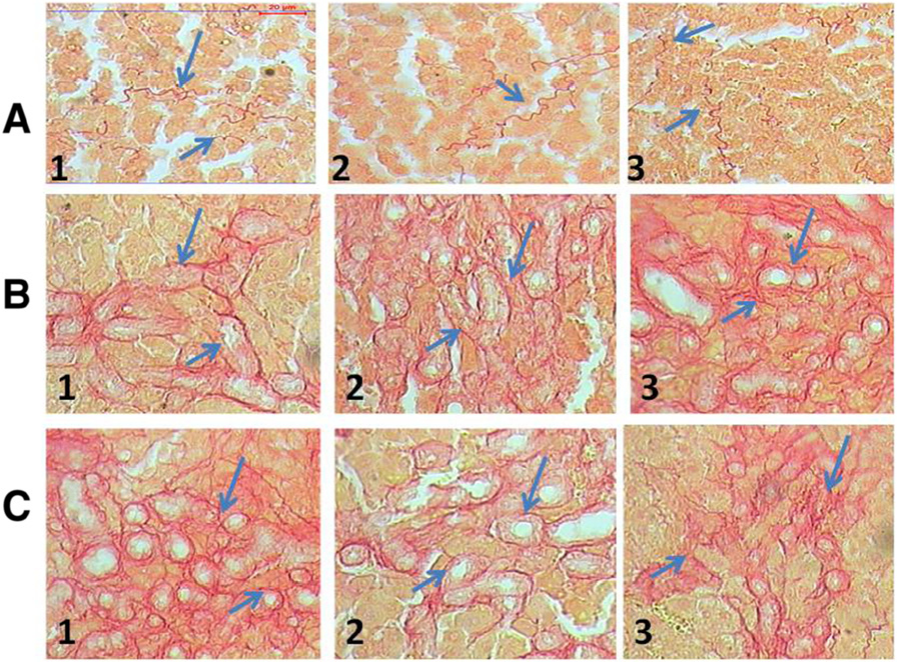
**Histological study of picrosirius red -stained liver sections (200×) of Sham, BDL and** ***Holothuria arenicola*** **extract (HaE) treated rats.** Rats were grouped: group 1, rats treated for 7 days; group 2, rats treated for 14 days and group 3, rats treated for 28 days. Liver sections from: **(A)** Control (Sham operated) rats; **(B)** bile duct-ligated rats; **(C)** bile duct-ligated rats and treated with *Holothuria arenicola* extract (HaE). Arrows indicated collagen deposition stained by picrosirius red.

**Figure 5:**
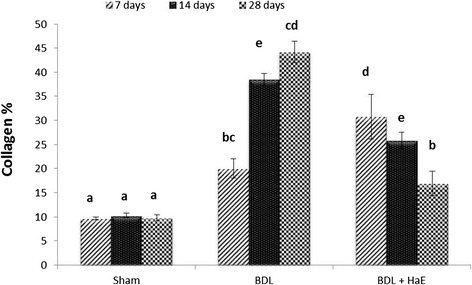
**Percentages of collagen deposition in Sham, BDL and** ***Holothuria arenicola*** **extract (HaE) treated rats for 7, 14 and 28 days.** Unshared letters between groups are the significance values at p < 0.05.

To assess the impact of HaE on hepatic fibrogenesis caused by BDL, liver sections were stained with picro-Sirius red for detecting the deposition of collagens. Compared with sections from the Sham control groups (Figure 4_A 1, 2_ &_3_), liver sections from BDL rats showed prominent red staining in the fibrotic septa between nodules (Figure 4_B 1, 2_ &_3_) suggesting a high level of collagen deposition. HaE treatment remarkably reduced the size stained with Sirius red in the liver (Figure 4_C 1, 2_ &_3_). BDL for 14 and 28 days recorded highest collagen percentages deposition 38.46 and 44.19%, respectively (Figure 5). Treatment with HaE for 28 days recorded a significant decrease (p < 0.05) in the collagen deposition percentage recording 19.91% (Figure 5).

### Efficiency of *Holothuria arenicola* extract on liver function enzymes, serum protein and serum albumin content

BDL group showed a significant increase (p < 0.05) in ASAT, ALAT and total protein levels following the three tested periods as compared to the corresponding Sham group (Table 1). While, serum albumin content decreased significantly (p < 0.05) after all tested periods. However, treatment with HaE significantly decreased (p < 0.05) serum ASAT and ALAT activities, but significantly increased serum albumin content after the three tested periods. The observed changes in the liver function enzymes after 28 days of treatment recorded the most improvement percentages than the other two tested periods. ASAT and ALAT levels were ameliorated by 77.86 and 70.15%, respectively, while the serum albumin content was ameliorated by 60.16%.

**Table 1:** **Effect of** ***Holothuria arenicola*** **extract on liver function enzymes and total protein level in serum of BDL rats**

**Parameters**	**Time period**	**Groups**			**% of improvement**
		**Sham**	**BDL**	**HaE**	
**ASAT (IU/L)**	7	149.70 ± 9.57^b^	191.55 ± 5.87^c^	147.25 ± 7.82^b^	29.59
	14	143.14 ± 9.08^b^	208.25 ± 10.02^c^	145.35 ± 10.4^b^	43.94
	28	144.90 ± 8.63^b^	262.85 ± 7.93^d^	150.02 ± 5.64^b^	77.86
**ALAT (IU/L)**	7	55.51 ± 0.99^a^	77.31 ± 5.39^b^	71.65 ± 4.27^b^	10.20
	14	54.18 ± 1.2^a^	103.86 ± 2.62^c^	67.95 ± 2.75^b^	66.27
	28	55.52 ± 1.1^a^	110.35 ± 5.25^c^	71.05 ± 5.45^b^	70.15
**Serum total protein (mg/100 ml)**	7	4.11 ± 0.46^a^	12.62 ± 0.5^c^	10.5 ± 1.47^b^	51.58
	14	4.15 ± 0.5^a^	14.86 ± 2.91^b^	12.95 ± 1.54^bc^	41.68
	28	4.17 ± 0.51^a^	15.42 ± 1.89^bc^	13.43 ± 0.84^bc^	47.72
**Serum albumin content (g/100 ml)**	7	5.51 ± 0.32^c^	2.81 ± 0.15^a^	3.51 ± 0.52^ab^	14.42
	14	5.86 ± 0.33^c^	3.84 ± 0.39^ab^	4.50 ± 0.22^b^	11.43
	28	6.1 ± 0.22^c^	2.63 ± 0.47^a^	6.30 ± 0.08^c^	60.16

Data are means ± SEM of six rats in each group.

Unshared letters between groups are the significance values at p < 0.05.

### Efficiency of *Holothuria arenicola* extract on serum cholestatic markers

As shown in Table 2, serum alkaline phosphatase activity, total bilirubin, direct bilirubin and indirect bilirubin levels were significantly higher in BDL rats as compared to their corresponding controls (*P* < 0.05). Administration of HaE to BDL group reduced ALP activity significantly (*P* < 0.05) after the three tested periods. Meanwhile, total bilirubin, direct bilirubin and indirect bilirubin decreased significantly (*P* < 0.05) following treatment with HaE for 7, 14 and 28 days. All studied cholestatic markers recorded highest improvement percentages following 28 days of HaE treatment.

**Table 2:** **Effect of** ***Holothuria arenicola*** **extract on serum cholestatic markers of BDL rats**

**Parameters**	**Time period**	**Groups**			**% of improvement**
		**Sham**	**BDL**	**HaE**	
**ALP (IU/L)**	7	149.49 ± 12.63^a^	218.29 ± 4.00^b^	208.51 ± 19.31^b^	6.5
	14	148.55 ± 12.88^a^	250.80 ± 26.41^b^	247.14 ± 4.84^b^	2.46
	28	149.75 ± 12.69^a^	247.18 ± 3.30^b^	153.70 ± 14.27^a^	62.40
**Total bilirubin (mg/100 ml)**	7	0.50 ± 0.08^a^	1.42 ± 0.67^b^	0.44 ± 0.8^a^	196
	14	0.48 ± 0.08^a^	1.30 ± 0.38^b^	0.43 ± 1.72 ^a^	181
	28	0.47 ± 0.08^a^	1.41 ± 0.72^b^	0.53 ± 0.42^a^	206
**Direct bilirubin (mg/100 ml)**	7	0.19 ± 0.02^a^	1.18 ± 0.4^b^	1.02 ± 0.38^a^	84.21
	14	0.20 ± 0.02^a^	1.45 ± 0.31^b^	1.10 ± 0.54^a^	175
	28	0.25 ± 0.02^a^	1.99 ± 0.17^b^	1.42 ± 0.30^a^	228
**Indirect bilirubin (mg/100 ml)**	7	0.24 ± 0.02^a^	1.3 ± 0.31^b^	1.09 ± 0.24^a^	87.5
	14	0.24 ± 0.02^a^	1.01 ± 0.46^b^	0.62 ± 0.76^c^	162.5
	28	0.28 ± 0.2^a^	1.61 ± 0.56^b^	0.98 ± 0.76^c^	225

Data are means ± SEM of six rats in each group.

Unshared letters between groups are the significance values at p < 0.05.

### The potency of *Holothuria arenicola* extract in improving the oxidative status of the liver

Malondialdehyde (MDA) and reduced glutathione (GSH) levels as well as glutathione-S-transferase (GST), catalase (CAT) and super oxide dismutase (SOD) activities in control, BDL and HaE administered rats were shown in Table 3.

**Table 3:** **Effect of** ***Holothuria arenicola*** **extract on the liver oxidative statues of BDL rats**

**Parameters**	**Time period**	**Groups**			**% of improvement**
		**Sham**	**BDL**	**HaE**	
**MDA (nmol/gm tissue)**	7	8.15 ± 0.57^bc^	8.79 ± 0.14^c^	8.10 ± 0.13^bc^	8.47
	14	8.13 ± 0.59 ^bc^	9.32 ± 0.37^c^	7.38 ± 0.46^ab^	23.86
	28	8.13 ± 0.57^bc^	15.04 ± 0.42^d^	6.77 ± 0.60^a^	101.72
**GSH (mg/gm tissue)**	7	7.79 ± 0.91^b^	6.65 ± 0.21^ab^	7.7 ± 0.68^b^	13.47
	14	7.84 ± 0.87^b^	5.28 ± 0.22^a^	8.41 ± 0.62^b^	39.79
	28	7.84 ± 0.89^b^	4.77 ± 0.62^a^	7.64 ± 0.39^b^	36.61
**CAT (U/min)**	7	2.10 ± 0.19^c^	0.60 ± 0.03^b^	2.75 ± 0.31^ac^	102
	14	2.09 ± 0.26^c^	0.56 ± 0.03^b^	2.47 ± 0.52^ac^	92
	28	2.08 ± 0.17^c^	0.16 ± 0.08^b^	1.66 ± 0.28^ab^	72
**GST (nmol/min/gm tissue)**	7	0.05 ± 0.003^c^	0.03 ± 0.002^b^	0.04 ± 0.003^ac^	20
	14	0.04 ± 0.008^cd^	0.03 ± 0.002^b^	0.04 ± 0.005^ac^	25
	28	0.05 ± 0.004^c^	0.02 ± 0.006^a^	0.05 ± 0.002^bc^	60
**SOD** **(U/gm tissue)**	7	64.68 ± 2.18^d^	52.61 ± 4.83^bc^	54.87 ± 4.14^bcd^	3.94
	14	67.22 ± 1.94^d^	39.66 ± 2.56^a^	47.401 ± 3.23^ab^	11.52
	28	60.43 ± 1.28^cd^	38.54 ± 3.15^a^	60.81 ± 1.33^cde^	36.85

Data are means ± SEM of six rats in each group.

Unshared letters between groups are the significance values at p < 0.05.

MDA levels were assessed as an indicator of lipid peroxidation. The liver MDA was found to be higher in the BDL group compared to their corresponding Sham-operated control groups following all tested periods, but this increase was significant (*P* < 0.05) only after 28 days of bile duct ligation. Treatment with HaE significantly decreased (*P* < 0.05) the MDA levels following 14 and 28 days of treatment (Table 3). However, the maximum improvement percentage was recorded following 28 days of HaE treatment.

The hepatic GSH level showed a marked reduction in the BDL group (*P* < 0.05) compared to their corresponding Sham-operated control group. However, this reduction was significantly (*P* < 0.05) following 14 and 28 days after bile duct ligation. Administration of HaE for 14 and 28 days significantly (*P* < 0.05) increased the level of GSH as compared to their corresponding BDL group (Table 3). The highest improvement percentage was recorded following 14 days of HaE treatment.

Bile duct ligation significantly (*P* < 0.05) decreased the level of CAT in the liver tissues in all tested groups, as compared to their corresponding controls (Table 3). However, treatment with HaE at 7, 14 and 28 days significantly (*P* < 0.05) increased the levels of CAT, as compared to the time matched BDL groups.

As shown in Table 3, bile duct ligation significantly decreased (*P* < 0.05) GST activity, as compared to their time matched sham groups following all tested time periods. Treatment with HaE for 7, 14 and 28 days caused significant increase (*P* < 0.05) in the GST activities as compared with their corresponding BDL groups.

Concerning the effect of bile duct ligation on the SOD activity, bile duct ligation significantly (*P* < 0.05) decreased the level of SOD in the liver tissues in all the tested groups, as compared to their corresponding controls (Table 3). However, treatment with HaE at all tested periods significantly (*P* < 0.05) increased the levels of SOD as compared to their time matched BDL groups. The highest improvement percentage was recorded following 14 days of HaE treatment.

## Discussion

Chronic liver disease is an important cause of morbidity and mortality and represents a major health problem worldwide [39]. The high prevalence of chronic liver diseases in Egypt has led to increasing numbers of Egyptian patients suffering from end-stage liver disease [40,41]. For the therapeutic strategies of liver injury and disease, it is important to find an antioxidant compound that is able to block liver injuries through the free radicals generated through toxic chemicals. During the course of evolution, many invertebrates have established as a selective advantage by endogenous production of protective chemicals [42]. The sea cucumber (*Holothuria*) is a marine invertebrate of the phylum Echinoderm and the class Holothuroidea found on the sea floor worldwide [43]. Recently, Esmat et al. [21] studied the antioxidant potential, and hepatoprotective activity of sea cucumber (*Holothuria atra*) against thioacetamide intoxication in rats. So, the present study aimed to investigate the therapeutic effect of phosphate buffer extract of the sea cucumber *Holothuria arenicola* in bile duct ligated rats.

Bile duct ligation model in rats has been used to study the mechanisms of new natural products in human patients with liver cirrhosis [44,45]. Phenolic-rich materials such as phytoplankton and particles derived from degrading marine macro-algae are the main sources of food for sea cucumbers, that can account for the presence of the active phenolic compounds in the body wall of sea cucumbers [43]. Phenolic compounds are very important because of their antioxidant activity [46]. The antioxidant activity of phenolic compounds is mainly attributable to their redox properties that play an important role as free radical scavengers, reducing agents, quenchers of singlet oxygen and complexes of pro-oxidant metals [47]. High-performance liquid chromatography analysis of the phenolic compounds in the HaE revealed the presence of 89.66% of chlorogenic acid. Chlorogenic acid is one of the most abundant polyphenols in the human diet and its potential hepatoprotective effect in several animal models of liver injury was reported [48].

Bile duct ligation induces a kind of liver fibrosis, that etiologically and pathogenitically resembles the biliary fibrosis in the human beings and is shown to induce cholestasis-related liver function impairments [49]. The most remarkable pathological characteristics of BDL -induced hepatotoxicity is increased production and deposition of extracellular matrix (ECM) component that accompanies most chronic liver disorders and its presence is a major factor contributing to hepatic failure [50]. The continuous accumulation of the ECM causes hepatofibrosis. Histopathological finding in the present study affirmed the study of Salas et al. [51] and showed that fibrosis leads to loss of normal architecture of the liver. The present study extended to confirm the finding of Friedman [52], who reported that collagen is the main component of the extracellular matrix in the fibrotic tissue. Preclinical studies have identified many potential therapies for fibrosis. These include interruption of matrix deposition and hence inhibition of collagen synthesis [53]. The extent of collagen contents assessed by the many fibrous septa within each microscopic field of liver tissues. The present study demonstrated an apparent arrest in the progression of collagen deposition in the HaE treated animals that can be a consequence of the increased mass of regenerated liver cells. This gives an additional support that HaE is able to condition the hepatocytes, accelerates regeneration of parenchyma cells, protects against membrane fragility and hence decreases leakage of the enzymes into circulation.

In BDL model, the hepatocellular excretion of bile constituents is markedly impaired eliciting its retention within hepatocytes. Cholestasis syndrome includes liver function disorder attributable to the obstruction of bile drainage into the intestine, with the consequent retention of bile constituents in the liver and their regurgitation in the blood [54,55]. In the condition of cholestasis, primarily hydrophobic bile salts and bilirubin have the most significant toxic effects [56]. In conjunction with the report of Zajic et al. [56] and Kim et al. [57], data from the present investigation showed that, the most important and reliable biochemical indicators of bile flow interruption were the increased values of bilirubin. In the present study, the maximum increase in conjugated (direct) bilirubin was recorded following 7 days. This result confirmed the finding of Zajic et al. [56], who reported that, the predominant hyper bilirubinaemia is always conjugated at the beginning of cholestasis when the damages are smaller. As the cholestasis dominates, a part of the direct bilirubin moves into blood plasma leads to hyperbilirubinemia of conjugated type [55]. The increased value of direct bilirubin in plasma following BDL may be due to a consequence of the increased concentration gradient between the cells and plasma or flowing out of bilirubin attributable to cell damages caused by obstruction in the bile flow [56,55].

Histological alterations and an increase in alkaline phosphatase (ALP) levels confirmed the damage produced by the bile duct ligation in rats [58]. In consonance with the findings of Nasehi et al. [49] and Kim et al. [57], the data recorded in the present study showed significant enhancement of the ALP activity following BDL in rats. This increase may be attributable to the retention of bile salts that damaged the membrane and consequently leads to the passing of the ALP enzyme into circulation [59,60]. It is known that, liver and bile duct disorders are followed by increased activity of ALP which is especially characteristic of the cholestastic syndrome [56]. Moreover, it has already been proved that in cholestasis the bile salts induce synthesis of new molecules of ALP [61]. The present study showed that treatment with HaE for 7 and 14 days failed to normalize the ALP level. Viewed in conjunction with the finding of Zajic et al. [56], alkaline phosphatase takes much longer to return to normal values and sometimes remains permanently increased despite the clinically successful biliary reconstruction. On the other hand, a decrease in collagen deposition and a reduction in ALP levels in the HaE group after 28 days of treatment suggest a better outcome in the treated groups.

In the assessment of liver damage, the determination of enzyme levels, such as serum aminotranferases, ASAT and ALAT usually used for the accurate detection and early diagnosis. The present study confirmed the finding of Olteanu et al. [62], who explained elevation in the serum enzymes ASAT and ALAT to the increase in hepatic cell membrane fluidity that led to enzyme release into circulation. In accord with the finding of Esmat et al. [21], the administration of HaE substantially attenuated the flares of the hepatic enzymes as evident by 77.86, 70.15 and 47.72% improvement in the activities of serum ASAT, ALAT and ALP respectively, in the BDL treated rats indicating maintenance of functional integrity of hepatic cell membrane.

Albumins as negative inflammatory reactants and markers of impaired synthetic liver function were significantly lower in patients with extrahepatic cholestasis [55]. In accord with the studies of Zajic et al. [56], the present study showed significant decrease in the albumin contents following the three tested periods after BDL in rats. It was reported that hypoalbuminaemia is most frequent in the presence of advanced chronic liver diseases [42]. The reduction in the albumin content in the present investigation may be attributable to significant damage of synthetic function of the liver resulting in decreased capability of the liver to synthesize albumins, that is manifested by the inverse correlation between the values of albumins and the increase of direct bilirubin. However, treatment of BDL- rats with HaE for 28 days bring back the level of albumin near to normal levels. Stimulation of albumin synthesis has been advanced as a contributory hepatoprotective mechanism, that accelerates the regeneration process and the production of liver cells [63]. Enhancement of the total protein content can be deemed as a useful index of the severity of cellular dysfunction in liver diseases as clearly shown in our studies. In consonance with the finding of Sharma and Shukla [64], the present study showed a significant increase in the total protein following BDL that may be a contributory self-healing mechanism that accelerates liver regeneration process. It was reported that liver dramatically increases the synthesis of certain proteins during the acute-phase response to the stress of the BDL [65]. Furthermore, systemic endotoxemia that frequently follows BDL could lead to increased neutrophilic response and increasing circulating visceral proteins [66].

Oxidative stress in cholestatic liver disease is a systemic phenomenon [67]. It was reported that oxidative stress occurs during cholestasis plays a role in cholestasis induced liver injury [68,39]. In accord with the reports of Kim et al. [57], the intensity of oxidative stress measured as an increase in the levels of lipid peroxidation end product, malondialdehyde (MDA) in the liver of BDL rats following all experimental periods. The elevation in MDA suggests enhanced lipid peroxidation leading to tissue damage and failure of antioxidant defense mechanisms to prevent formation of excessive free radicals. The underlying mechanisms of increased systemic oxidative stress during cholestasis may be attributable to the retention of toxic bile acids, that stimulate the generation of reactive oxygen species (ROS) in hepatocytes which stimulate lipid peroxidation [10]. Moreover, Schmucker et al. [69] have shown that the hydrophobic bile acids damaged the hepatocelular membrane attributable to their detergent-like influence which confirmed in the present investigation by the histopathological examination. In conjunction with the reports of Damnjanović et al. [55], the present study showed positive correlation between oxidative stress and hyper bilirubinaemia noticed in the present study, which may be considered as a form of protective effect [55]. However, the decrease of lipid peroxidation-mediated oxidative stress may be a potential and effective strategy for the prevention and treatment of hepatic failure [70]. The present study affirmed the finding of Esmat et al. [21], who reported that sea cucumber body wall extract significantly decreased MDA levels in injured liver tissues, suggesting that the antifibrotic mechanism of HaE may be attributable to its phenolic antioxidant effect.

Glutathione reduced (GSH), a key antioxidant, is an important constituent of intracellular protective mechanisms against various noxious stimuli including oxidative stress [71]. Because of their exposed sulfhydryl groups, non-protein sulfhydryls bind a variety of electrophilic radicals and metabolites that may be damaging to the cells [72]. Reduced amount of glutathione in patients with cholestasis decrease hepatobiliary transport of toxic organic components leading to the development of complications accompanying cholestasis [73]. Insufficiency in non-enzymatic antioxidant GSH following BDL could be the consequence of increased utilization for trapping free radicals. In accordance with the report of Þener et al. [71] and Kim et al. [57], our results also support the notion that depletion of tissue GSH as observed in the BDL-induced hepatic injury is one of the major factors that permit lipid peroxidation and subsequent tissue damage. Treatment with HaE in the present study restored GSH content following the three tested periods. In accord with our results, Gaté et al. [74] and Fahmy & Hamdi [42] have reported that dietary supplementation of the marine extract of the *Crassostrea gigas* clams and mantis shrimp *Erugosquilla massavensis* increased GSH level in the liver of rats. This increase is a reflection of increased synthesis of GSH in the liver [75].

Viewed in conjunction with the finding of Somi et al. [76], the present study demonstrated that bile duct ligation usually decreases antioxidant enzyme (GST, SOD and CAT) activities in hepatic tissue that may be attributable to mitochondrial toxicity induced by intrahepatocyte concentration of biliary acids in chronic cholestasis. In accord with our results, Sanzgiri et al. [77] have reported that the enhanced free radical concentration resulting from the oxidative stress conditions can cause loss of enzymatic activity. Moreover, the enhancements in antioxidant enzyme activities may prevent the accumulation of excessive free radicals and protect liver following BDL. Tissues and cells would be subjected to oxidative injuries when large quantities of inner free radicals are generated or the activities of antioxidant system deteriorate. Accordingly, antioxidant therapy represents a potential strategy to prevent liver injury and fibrosis. Treatment with HaE normalized the antioxidant levels through their rich of polyphenolic compound especially chlorogenic acid that has the ability to scavenge free radicals.

## Conclusion

The present prospective study serves to extend the growing number of our earlier investigations on therapeutic products from aquatic sources and confirm that HaE improved antioxidant status, and thereby prevented liver damage following cholestasis. The antifibrotic effect of HaE produced maximum improvement following 28 days of treatment that may be attributable to its phenolic compounds specially chlorogenic acid.
